# The causal role of immune cells on lung cancer: a bi-directional Mendelian randomization (MR) study

**DOI:** 10.18632/aging.205917

**Published:** 2024-06-12

**Authors:** Hongyu Zhu, Caihua Chen, Haixie Guo, Bo Zhang, Quanteng Hu

**Affiliations:** 1 Department of Thoracic Surgery, Taizhou Hospital of Zhejiang Province Affiliated to Wenzhou Medical University, Taizhou 317000, Zhejiang, China

**Keywords:** lung cancer, immune cells, Mendelian randomization, causality

## Abstract

Immune cells play a vital role in the development and progression of lung cancer (LC). We aimed to explore the causal role of immune cells in LC with Mendelian randomization (MR) study. Summary statistic data used in the study were obtained from genome-wide association studies (GWAS). A comprehensive two-sample MR was carried out to explore the causal role of 731 immune cell traits (ICTs) in LC, Non-small cell lung cancer (NSCLC), and Small cell lung cancer (SCLC). An inverse-variance weighted (IVW) approach was applied to present the MR estimates. The heterogeneity test was performed using Cochran’s Q statistic. MR-Egger intercept test and MR-PRESSO were utilized for the pleiotropy test. MR showed that 15, 31, and 11 ICTs had protective effects on LC, NSCLC, and SCLC, respectively, and 12, 31, and 11 ICTs had adverse effects on LC, NSCLC, and SCLC, respectively. Of note, CD3 on CD28^+^ CD4^+^ in the Treg panel could significantly increase the risk of LC, as well as NSCLC and SCLC. Moreover, the MR results revealed that LC was vital in IgD on IgD^+^ in the B cell panel and NSCLC on CCR2 on CD14- CD16- in the Monocyte panel. Our study revealed multiple close connections between immune cells and LC.

## INTRODUCTION

Globally, lung cancer (LC) is the most common cancer to be diagnosed and the leading cause of cancer-related death [1, 2]. It is estimated that over 230,000 new cases are diagnosed every year, and more than 180,000 people die of LC worldwide [3]. LC contains two histological subtypes: Small cell lung cancer (SCLC) and Non-small cell lung cancer (NSCLC) [4], accounting for 15% and 85% of all LC cases, respectively [4, 5].

Tumor growth and progression are significantly influenced by the immune system. Immune cells provide the function of immune surveillance to recognize and eliminate cancer cells [6]. The immune system recognizes the mutated cells and the metastatic tumor cells in the body and directly kills tumor cells by releasing cytotoxics, cytokines, and other methods to block the development and metastasis of tumors. In addition, immune cells could regulate the activity of the immune system, enhance immune response, and thus enhance immune attack against tumors [7, 8]. At the same time, immune cells can also inhibit the immune escape ability of tumor cells, preventing them from attacking the immune system [9]. Nevertheless, malignant cells can avoid tumor-related antigens recognized by immune cells in various ways, promoting their development, infiltration, and metastasis [10, 11]. It is precisely based on the above mechanisms that researchers have invented a novel therapy for numerous tumors, anti-PD1/L1 immune checkpoint inhibitor (ICI), against tumors acting by harnessing the immune system [12]. The combination of ICI and chemotherapy has become a first-line treatment strategy for advanced-stage LC patients. However, even today, only a limited number of LC patients have sustained benefits from ICI [13].

In epidemiology, Mendelian randomization (MR) is an analytical tool used to investigate etiological relationships between risk variables (exposure) and outcomes [14]. In MR, instrumental variables (IVs) of genetic variation for exposure could avoid the interference of confounding factors common in observational studies and high reliability and practicality [15].

In the study, single nucleotide polymorphism (SNP) was gathered from genome-wide association studies (GWAS; https://gwas.mrcieu.ac.uk/) to present IVs. Then, we determined the causal role of immune cell traits (ICTs) on LC with a comprehensive two-sample MR analysis.

## RESULTS

### The causal effect of ICTs on LC

**Figure 1:**
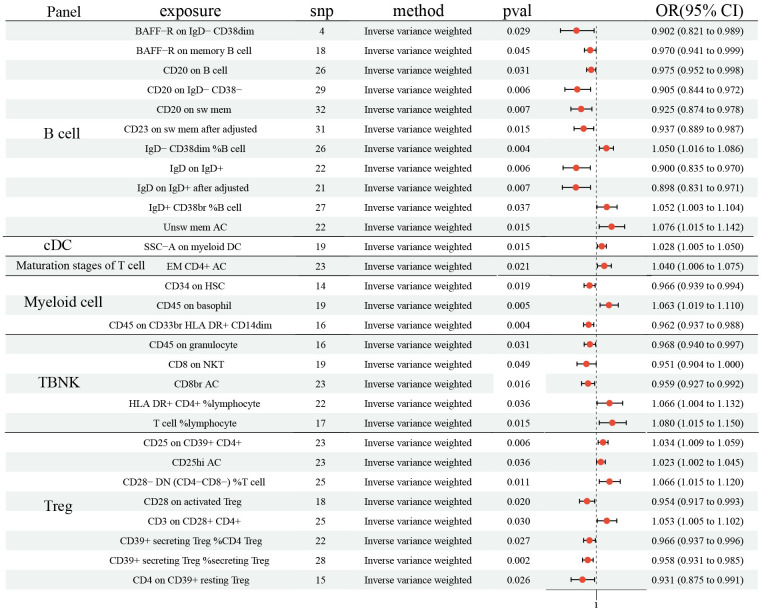
**Forest plots showed the causal role of immune cell traits on LC.** CI: confidence interval; OR: odds ratio; LC: Lung cancer.

In the study, the protective effects of 15 ICTs on LC were found with IVW method (BAFF−R on IgD^−^ CD38dim (odds ratio (OR): 0.902, 95% CI [0.821, 0.989], P= 0.029); BAFF−R on memory B cell (OR: 0.970, 95% CI [0.941, 0.999], P= 0.031); CD20 on B cell (OR: 0.975, 95% CI [0.952, 0.998], P= 0.031); CD20 on IgD^−^ CD38^−^ (OR: 0.905, 95% CI [0.844, 0.972], P= 0.006); CD20 on sw mem (OR: 0.925, 95% CI [0.874, 0.978], P= 0.007); IgD on IgD^+^ (OR: 0.900, 95% CI [0.835, 0.970], P= 0.006); IgD^+^ CD34 on HSC (OR: 0.966, 95% CI [0.939, 0.994], P= 0.019); CD45 on CD33br HLA DR^+^ CD14dim (OR: 0.962, 95% CI [0.937, 0.988], P= 0.004); CD45 on granulocyte (OR: 0.968, 95% CI [0.940, 0.997], P= 0.031); CD8 on NKT (OR: 0.951, 95% CI [0.904, 1.000], P= 0.049); CD8br AC (OR: 0.959, 95% CI [0.927, 0.992], P= 0.016); CD28 on activated (Regulatory T cells) Treg (OR: 0.954, 95% CI [0.917, 0.993], P= 0.020); CD39^+^ secreting Treg% CD4 Treg (OR: 0.966, 95% CI [0.937, 0.996], P= 0.027); CD39^+^ secreting Treg% secreting Treg (OR: 0.958, 95% CI [0.931, 0.985], P= 0.002); CD4 on CD39^+^ resting Treg (OR: 0.931, 95% CI (0.875, 0.991), P= 0.026), Figure 1). Additionally, 12 ICTs had adverse effects on LC (IgD^−^ CD38dim% B cell (OR: 1.050, 95% CI [1.016, 1.086], P= 0.004); IgD^+^ CD38br %B cell (OR: 1.052, 95% CI [1.003, 1.104], P= 0.037); Unsw mem AC (OR: 1.076, 95% CI [1.015, 1.142], P= 0.004); SSC−A on myeloid DC (OR: 1.028, 95% CI [1.005, 1.050], P= 0.015); EM CD4^+^ AC (OR: 1.040, 95% CI [1.006, 1.075], P= 0.021); CD45 on basophil (OR: 1.063, 95% CI [1.019, 1.110], P= 0.005); HLA DR^+^ CD4^+^% lymphocyte (OR: 1.066, 95% CI [1.004, 1.132], P= 0.036); T cell^%^ lymphocyte (OR: 1.080, 95% CI [1.015, 1.150], P= 0.015); CD25 on CD39^+^ CD4^+^ (OR: 1.034, 95% CI [1.009, 1.059], P= 0.006); CD25hi AC (OR: 1.023, 95% CI [1.002, 1.045], P= 0.004); CD28^−^ DN (CD4^−^CD8^−^)% T cell (OR: 1.066, 95% CI [1.015, 1.120], P= 0.004); CD3 on CD28^+^ CD4^+^ (OR: 1.053, 95% CI [1.005, 1.102], P= 0.026), Figure 1). Among these 731 ICTs, heterogeneity was detected in 4 ICTs: CD20 on IgD^-^ CD38^-^ (MR Egger P= 0.018; IVW P= 0.007); CD20 on sw mem (MR Egger P= 0.007; IVW P= 0.008); IgD on IgD^+^ (MR Egger P= 0.030; IVW P= 0.012); CD8 on NKT (MR Egger P= 0.045; IVW P= 0.045) (Supplementary Table 1). The Egger intercept test did not show horizontal pleiotropy (Supplementary Table 2). However, MR-PRESSO presented horizontal pleiotropy in CD20 on sw mem (P= 0.015) and IgD on IgD^+^ (P= 0.015) (Supplementary Table 2). Then, we removed outlier SNPs and re-analyzed the causal effect: CD20 on sw mem (OR: 0.937, 95% CI [0.889, 0.987], P= 0.015); IgD on IgD^+^ (OR: 0.898, 95% CI [0.831, 0.971], P= 0.007). The MR-PRESSO was not statistically significant: CD20 on sw mem (P= 0.077) and IgD on IgD^+^ (P= 0.140) (Supplementary Table 2). The leave-one-out analysis showed no SNP could drive the causal estimates.

### The causal effect of ICTs on NSCLC

**Figure 2:**
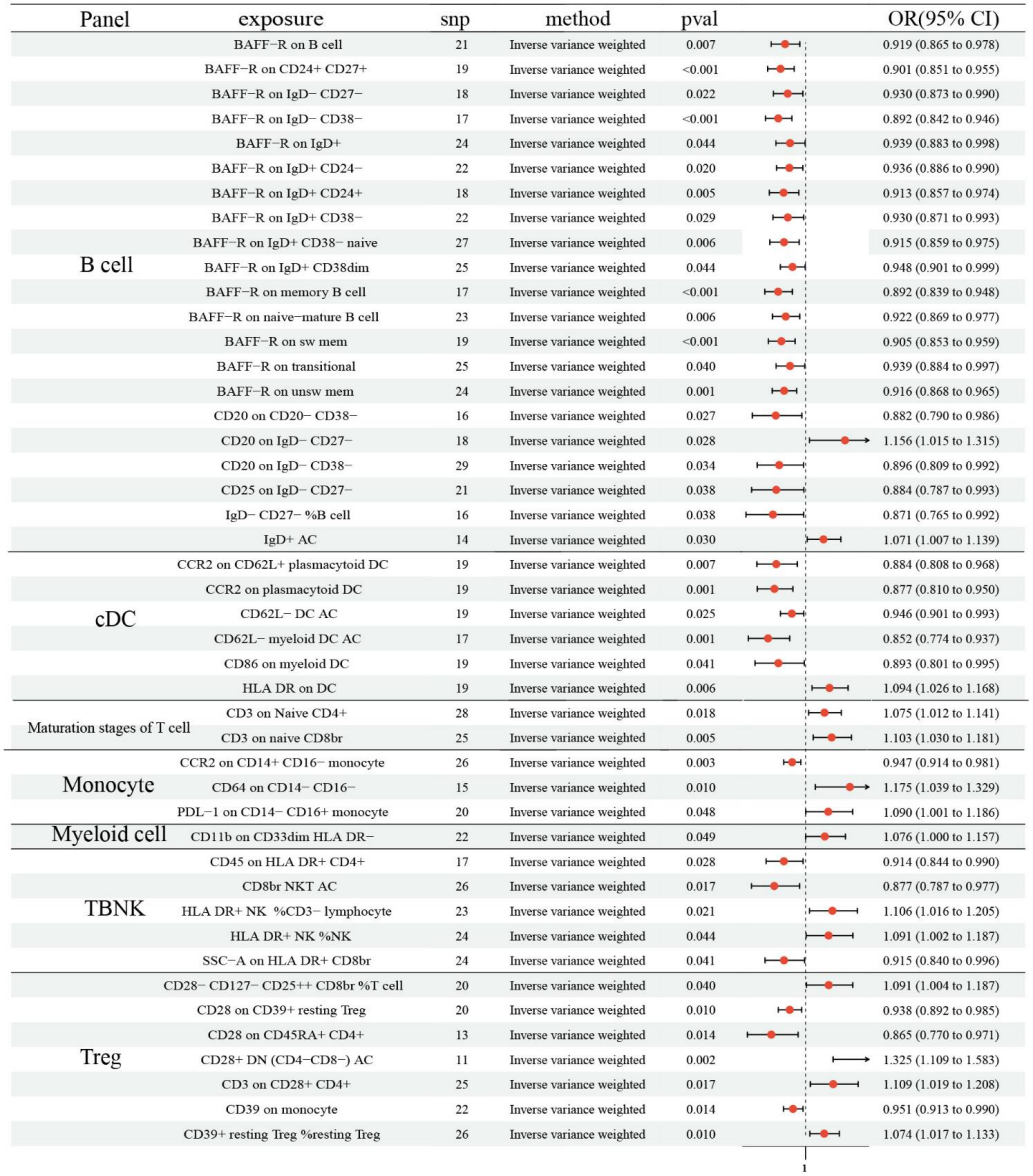
**Forest plots showed the causal role of immune cell traits on NSCLC.** CI: confidence interval; OR: odds ratio; NSCLC; Non-small cell lung cancer.

As shown in Figure 2, 21 ICTs had vital role on NSCLC at B cell panel: BAFF-R on B cell (OR: 0.919, 95% CI [0.865, 0.978], P= 0.007); BAFF-R on CD24^+^ CD27^+^ (OR: 0.901, 95% CI [0.851, 0.955], P <0.001); BAFF-R on IgD^-^ CD27^-^ (OR: 0.930, 95% CI [0.873, 0.990], P=0.022); BAFF-R on IgD^-^ CD38^-^ (OR: 0.892, 95% CI [0.842, 0.946], P <0.001); BAFF-R on IgD^+^ (OR: 0.939, 95% CI [0.883, 0.998], P=0.044); BAFF-R on IgD^+^ CD24- (OR: 0.936, 95% CI [0.886, 0.990], P=0.020); BAFF-R on IgD^+^ CD24^+^ (OR: 0.913, 95% CI [0.857, 0.974], P=0.005); BAFF-R on IgD^+^ CD38- (OR: 0.930, 95% CI [0.871, 0.993], P=0.029); BAFF-R on IgD^+^ CD38- naïve (OR: 0.915, 95% CI [0.859, 0.975], P= 0.006); BAFF-R on IgD^+^ CD38dim (OR: 0.948, 95% CI [0.901, 0.999], P= 0.044); BAFF-R on memory B cell (OR: 0.892, 95% CI [0.839, 0.948], P <0.001); BAFF-R on naive-mature B cell (OR: 0.922, 95% CI [0.869, 0.977], P= 0.006); BAFF-R on sw mem (OR: 0.905, 95% CI [0.853, 0.959], P <0.001); BAFF-R on transitional (OR: 0.939, 95% CI [0.884, 0.997], P= 0.040); BAFF-R on unsw mem (OR: 0.916, 95% CI [0.868, 0.965], P= 0.001); CD20 on CD20^-^ CD38^-^ (OR: 0.882, 95% CI [0.790, 0.986], P= 0.027); CD20 on IgD^-^ CD27^-^ (OR: 1.156, 95% CI [1.015, 1.315], P= 0.028); CD20 on IgD^-^ CD38^-^ (OR: 0.896, 95% CI [0.809, 0.992], P= 0.034); CD25 on IgD^-^ CD27^-^ (OR: 0.884, 95% CI [0.787, 0.993], P= 0.038); IgD^-^ CD27^-^ %B cell (OR: 0.871, 95% CI [0.765, 0.992], P= 0.038); IgD^+^ AC (OR: 1.071, 95% CI [1.007, 1.139], P= 0.030). In addition, 6 ICTs in cDC panel (CCR2 on CD62L^+^ plasmacytoid DC (OR: 0.884, 95% CI (0.808, 0.968), P= 0.007); CCR2 on plasmacytoid DC (OR: 0.877, 95% CI [0.810, 0.950], P= 0.001); CD62^L-^ DC AC (OR: 0.946, 95% CI [0.901, 0.993], P= 0.025); CD62L^-^ myeloid DC AC (OR: 0.852, 95% CI [0.774, 0.937], P= 0.001); CD86 on myeloid DC (OR: 0.893, 95% CI [0.801, 0.995], P= 0.041); HLA DR on DC (OR: 1.094, 95% CI [1.026, 1.168], P= 0.006)) and 3 in Monocyte (CCR2 on CD14^+^ CD16- monocyte (OR: 0.947, 95% CI [0.914, 0.981], P= 0.003); CD64 on CD14^-^ CD16^-^ (OR: 1.175, 95% CI [1.039, 1.329], P= 0.010); PDL-1 on CD14^-^ CD16^+^ monocyte (OR: 1.090, 95% CI [1.001, 1.186], P= 0.048)) also had causal effect NSCLC. 2 ICTs in Maturation stages of T cell panel (CD3 on Naive CD4^+^ (OR: 1.075, 95% CI [1.012, 1.141], P= 0.018); CD3 on naive CD8br (OR: 1.103, 95% CI [1.030, 1.181], P= 0.005)) were disadvantageous factors for NSCLC. Similar result was observed in CD11b on CD33dim HLA DR- (OR: 1.076, 95% CI [1.000, 1.186], P= 0.048). There were 5 and 7 ICTs in TBNK and Treg panel identified, respectively (TBNK panel: CD45 on HLA DR^+^ CD4^+^ (OR: 0.914, 95% CI [0.844, 0.990], P= 0.028); CD8br NKT AC (OR: 0.877, 95% CI [0.787, 0.977], P= 0.017); HLA DR^+^ NK% CD3- lymphocyte (OR: 1.106, 95% CI [1.016, 1.205], P= 0.021); HLA DR^+^ NK% NK (OR: 1.091, 95% CI [1.002, 1.187], P= 0.044); SSC-A on HLA DR^+^ CD8br (OR: 0.915, 95% CI [0.840, 0.996], P= 0.041); Treg panel: CD28^-^ CD127^-^ CD25^+^ CD8br% T cell (OR: 1.091, 95% CI [1.004, 1.187], P= 0.040); CD28 on CD39^+^ resting Treg (OR: 0.938, 95% CI [0.892, 0.985], P= 0.010); CD28 on CD45RA^+^ CD4^+^ (OR: 0.865, 95% CI [0.770, 0.971], P= 0.014); CD28^+^ DN (CD4-CD8-) AC (OR: 1.325, 95% CI [1.109, 1.583], P= 0.002); CD3 on CD28^+^ CD4^+^ (OR: 1.109, 95% CI [1.019, 1.208], P= 0.017); CD39 on monocyte (OR: 0.951, 95% CI [0.913, 0.990], P= 0.014); CD39^+^ resting Treg %resting Treg (OR: 1.074, 95% CI [1.017, 1.133], P= 0.010)). No horizontal pleiotropy and heterogeneity were found among 731 ICTs (Supplementary Tables 1, 2). Furthermore, no change of the causal estimates was found by removing a particular SNP.

### The causal effect of ICTs on SCLC

**Figure 3:**
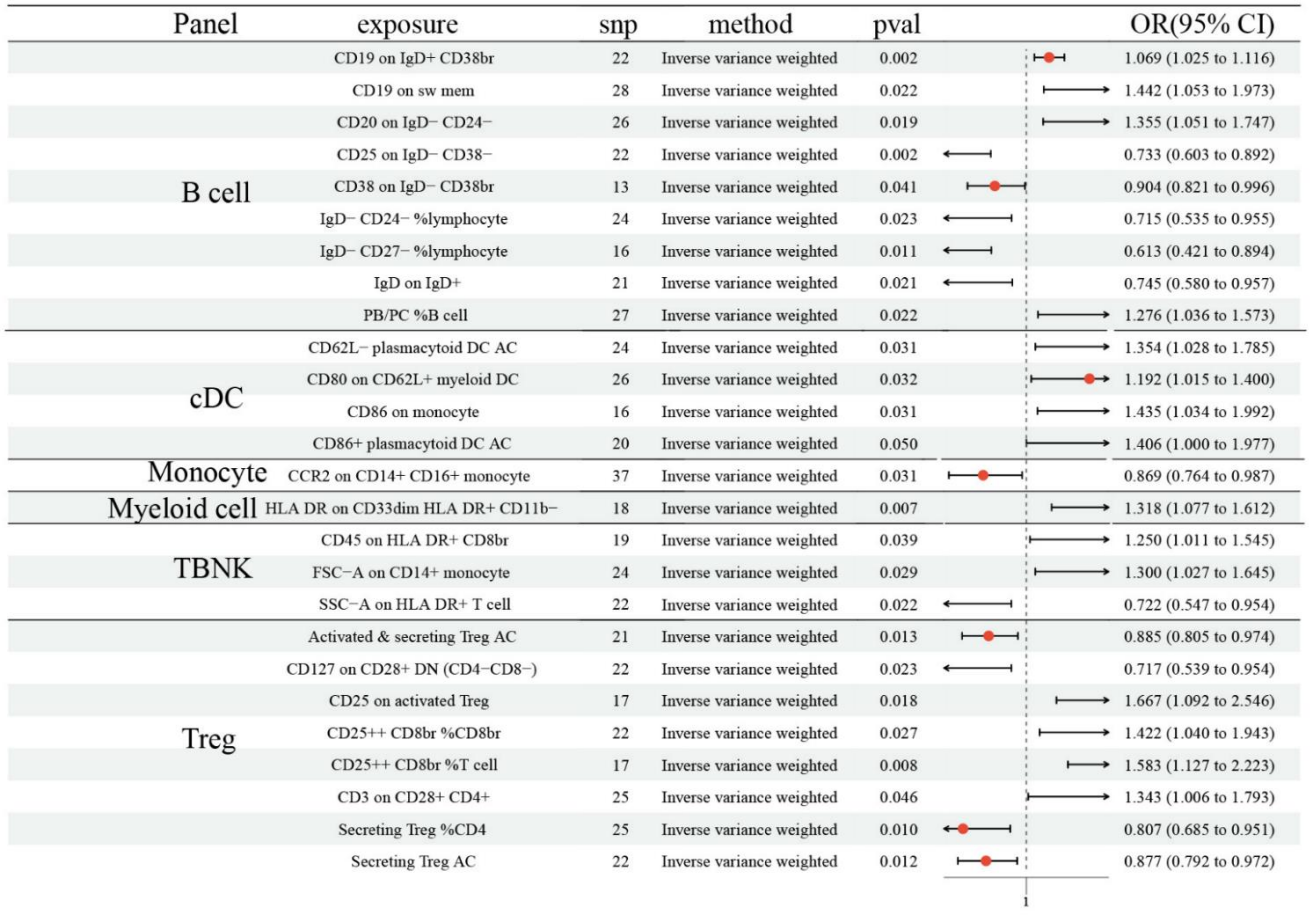
**Forest plots showed the causal role of immune cell traits on SCLC.** CI: confidence interval; OR: odds ratio; SCLC; Small cell lung cancer.

The MR demonstrated 15 ICTs would increase the risk of SCLC: CD19 on IgD^+^ CD38br (OR: 1.069, 95% CI [1.025, 1.116], P=0.002); CD19 on sw mem (OR: 1.442, 95% CI [1.053, 1.973], P=0.022); CD20 on IgD^-^ CD24^-^ (OR: 1.355, 95% CI [1.051, 1.747], P=0.019); PB/PC% B cell (OR: 1.276, 95% CI [1.036, 1.573], P=0.022); CD62L^-^ plasmacytoid DC AC (OR: 1.354, 95% CI [1.028, 1.785], P=0.031); CD80 on CD62L^+^ myeloid DC (OR: 1.192, 95% CI [1.015, 1.400], P=0.032); CD86 on monocyte (OR: 1.435, 95% CI [1.034, 1.992], P=0.031); CD86^+^ plasmacytoid DC AC (OR: 1.406, 95% CI [1.000, 1.977], P=0.050); HLA DR on CD33dim HLA DR^+^ CD11b- (OR: 1.318, 95% CI [1.077, 1.612], P=0.007); CD45 on HLA DR^+^ CD8br (OR: 1.250, 95% CI [1.011 1.545], P=0.039); FSC-A on CD14^+^ monocyte (OR: 1.300, 95% CI [1.027, 1.645], P=0.029); CD25 on activated Treg (OR: 1.667, 95% CI [1.092, 2.546], P=0.018); CD25^++^ CD8br% CD8br (OR: 1.422, 95% CI [1.040, 1.943], P=0.027); CD25^++^ CD8br% T cell (OR: 1.583, 95% CI [1.127, 2.223], P=0.008); CD3 on CD28^+^ CD4^+^ (OR: 1.343, 95% CI [1.006, 1.793], P=0.046) (Figure 3). Moreover, 11 ICTs would decrease the risk of SCLC: CD25 on IgD^-^ CD38^-^ (OR: 0.733, 95% CI [0.603, 0.892], P=0.002); CD38 on IgD^-^ CD38br (OR: 0.904, 95% CI [0.821, 0.996], P=0.041); IgD^-^ CD24^-^% lymphocyte (OR: 0.715, 95% CI [0.535, 0.955], P=0.023); IgD^-^ CD27^-^% lymphocyte (OR: 0.613, 95% CI [0.421, 0.894], P=0.011); IgD on IgD^+^ (OR: 0.745, 95% CI [0.580, 0.957], P=0.021); CCR2 on CD14^+^ CD16^+^ monocyte (OR: 0.869, 95% CI [0.764, 0.987], P=0.031); SSC-A on HLA DR^+^ T cell (OR: 0.722, 95% CI [0.547, 0.954], P=0.022); Activated and secreting Treg AC (OR: 0.885, 95% CI [0.805, 0.974], P=0.013); CD127 on CD28^+^ DN (OR: 0.717, 95% CI [0.539, 0.954], P=0.023); Secreting Treg% CD4 (OR: 0.807, 95% CI [0.685, 0.951], P=0.010); Secreting Treg AC (OR: 0.877, 95% CI [0.792, 0.972], P=0.012) (Figure 3). We did not determine any horizontal pleiotropy and heterogeneity among 731 ICTs (Supplementary Tables 1, 2).

### The causal effect of LC, NSCLC, and SCLC on ICTs

**Figure 4:**
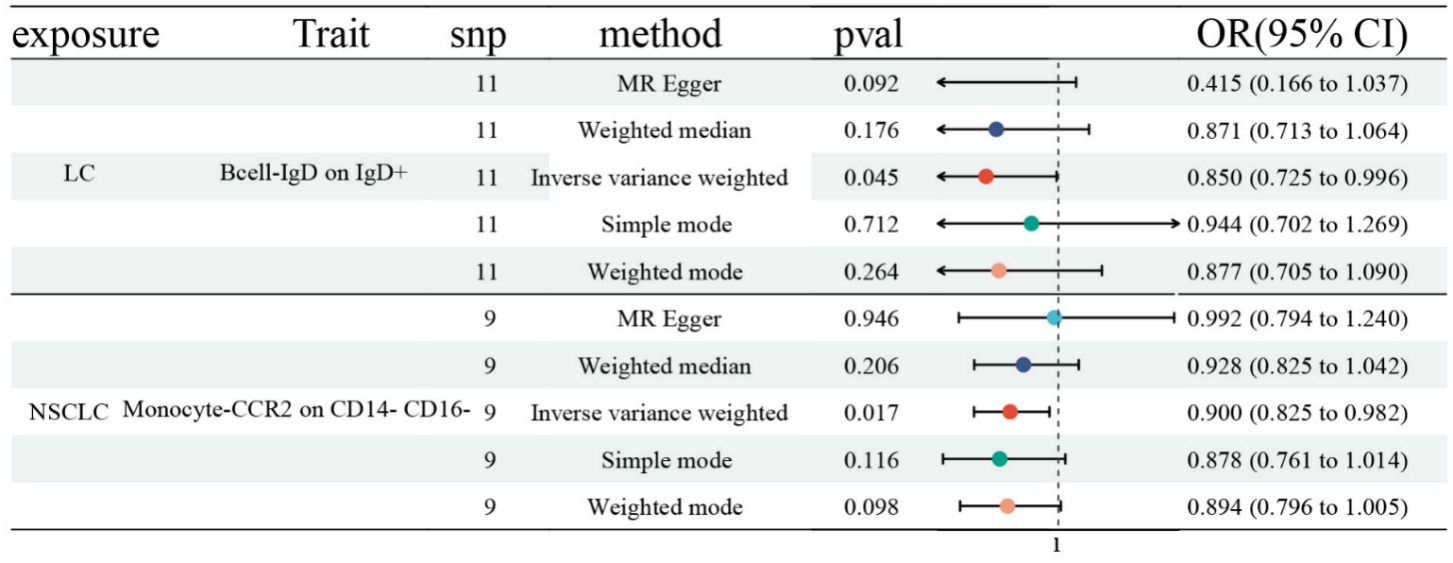
**Forest plots showed the causal role of LC, NSCLC, and SCLC on immune cell traits.** CI: confidence interval; OR: odds ratio; LC: Lung cancer; NSCLC; Non-small cell lung cancer; SCLC; Small cell lung cancer.

Finally, we explored the influences of LC, NSCLC, and SCLC on ICTs, which had a vital role on LC (27 ICTs), NSCLC (45 ICTs), and SCLC (26 ICTs). We first identified SNPs strongly associated with LC (NSCLC and SCLC) as genetic instruments for exposure. Then, we detected a significant effect of LC on IgD on IgD^+^ B cell (OR: 0.850, 95% CI [0.725, 0.996], P=0.045), as well as NSCLC on CCR2 on CD1^4-^ CD16^-^ Monocyte (OR: 0.900, 95% CI [0.825, 0.982], P=0.017) (Figure 4). However, no statistically significant causal effect of SCLC on ICTs was discovered. No horizontal pleiotropy and heterogeneity were detected (Supplementary Tables 1, 2).

## DISCUSSION

The immune system is an important consideration influencing the occurrence and development of LC [10]. Herein, we explored the risk coefficient of 731 ICTs on LC using two-sample MR. All data were gathered from a public database: GWAS.

We found the protective effects of 15 ICTs on LC, and the adverse effects of 12. MR-PRESSO hinted that horizontal pleiotropy exited in CD20 on sw mem and IgD on IgD^+^, although the Egger intercept test did not show horizontal pleiotropy. For correcting the possible pleiotropy that could substantially affect the estimation results, we removed SNP= rs9271146 in CD20 on sw mem and SNP= rs79925536 in IgD on IgD^+^. After the horizontal pleiotropic outlier, no horizontal pleiotropy was detected. In addition, MR analysis identified 31 and 11 ICTs that decreased the risk of NSCLC and SCLC, respectively. Moreover, the results indicated that 14 and 15 ICTs were unfavorable risk factors for NSCLC and SCLC, respectively. The heterogeneity and pleiotropy tests did not show positive results, ensuring the validity of the causal relationship conclusion.

Of note, CD3 on CD28^+^ CD4^+^ in the Treg panel could significantly increase the risk of LC, as well as NSCLC and SCLC. Treg cells are potent immunosuppressive cells important in maintaining immune homeostasis by regulating and suppressing immune reactions [16, 17]. Nevertheless, in several cancers, Treg could promote tumor progression because the recruited Treg cells in the tumor microenvironment contribute to cancer cells’ escape from immunological surveillance [18], which was in line with our finding. CD4+ were the main characteristic of Treg cells, born in the bone marrow and developed in the thymus [19]. CD28 is the most crucial co-stimulatory molecule, and the second signal can fully activate T cells, secrete cytokines, and express cytokine receptors [20]. CD3, an important leukocyte differentiation antigen, expressed on the surface of almost all T cells. When antigens bind to TCR, they promote the transmission of signals into cells and then trigger T cells differentiation and activation, the secretion of cytokines, and cell apoptosis [21]. However, in the study, CD3 on CD28^+^ CD4^+^ in Treg was a disadvantageous risk factor.

Finally, we determined the causal effects of LC, NSCLC, and SCLC on relevant ICTs. The MR results revealed that LC had a vital role in IgD on IgD^+^ in the B cell panel, along with NSCLC on CCR2 on CD14^-^ CD16^-^ in the Monocyte panel. IgD^+^ B cells predominantly co-express IgM and epsilon transcripts [22, 23]. CCR2 belongs to the family of G proteins and could be divided into two subtypes, CCR2A and CCR2B [21]. CCR2 expression on monocytes is critical for the recruitment of tumor-associated macrophages, inflammation, cancer growth, and metastasis [24]. By binding to CCL2, CCR2 regulates the expression of IL-1, TNF, and CCL2 to activate corresponding signaling pathways to produce cytokine and regulate cell growth, differentiation, and apoptosis [24].

Herein, we determined the causal role of 731 ICTs on LC with a MR design. The design can replicate randomized controlled trials at a reduced cost. Additionally, compared with observational studies, MR analysis could remove the reverse causal effect. Although the MR results showed a strong correlation between ICTs and LC, some limitations still need to be explained. 1) We performed the heterogeneity test and pleiotropy test. However, not all potential heterogeneity and horizontal pleiotropy can be eliminated. 2) All data were gathered from the GWAS database, leading to some clinical index missing, like age. A stratified analysis was needed. 3) All participants were from Europe. Hence, applying this conclusion to other racial groups may be limited. 4) Only 1627 NSCLC and 179 SCLC cases were collected in the study, which may bias the results.

It is the first time to analyze the causality from 731 ICTs on LC with the MR design. Our results revealed multiple close connections between immune cells and LC.

## MATERIALS AND METHODS

### Study design

Herein, the causal role of 731 ICTs on LC (as well as SCLC and NSCLC) was investigated with a two-sample MR analysis. Then, the role of LC on ICTs with a reverse MR analysis was also assessed. MR must follow three pivotal assumptions: 1) the selected IVs should be directly related to each immune cell signature; 2) No potential confounders correlated with IVs between exposure and outcome; 3) The IVs influence outcome only via exposure, rather than other pathways.

### GWAS data sources

GWAS data for LC, SCLC, and NSCLC were gathered from the Ebi-a-GCST90018875, Finn-b-C3_SCLC, and Finn-b-C3_LUNG_NONSMALL dataset, respectively [25]. 492 803 samples were in LC set: 3791 LC cases and 489012 control cases; 218 792 samples in SCLC set: 179 SCLC cases and 218 613 control cases; 218 792 samples in NSCLC set: 1627 NSCLC cases and 217 165 control cases. The original GWAS data 731 ICTs (Ebi-a-GCST0001391 to Ebi-a-GCST0002121) contained 3757 cases [23] and four trait types, absolute counts (AC), morphological parameters (MP), median fluorescence intensities (MFI), and relative counts (RC) in six panels: TBNK (B cells, natural killer cells, T cells), CDCs, mature stages of T cells, B cells, monocytes, Treg, and myeloid cells panels [26]. All participants are Europeans.

### Selection of IVs

A relaxed cut-off value (p < 1e×10^−5^) filtered out SNPs strongly associated with each immune cell trait [27]. A stricter threshold (p <5e×10^-8^) was used for determining SNPs representing LC, NSCLC, and SCLC [28]. Removing linkage disequilibrium (LD) with the cut-off value: R^2^ < 0.001 within 10000 kb clumping distance using the European reference panel of the 1000 Genome Project. Then, we calculated the F-statistics (per SNP by squaring the beta divided by the standard error). When an SNP with F-statistic > 10, it indicated this was a sufficiently strong instrument for exposure and would be selected. 13318 SNPs were determined as IVs of 731 ICTs (Supplementary Table 3). In addition, 11, 10, and 9 SNPs were selected as IVs of LC, SCLC, and NSCLC, respectively (Supplementary Table 3).

### Statistical analysis

R 4.3.1 (https://www.r-project.org) was used for all statistical analyses. MR analysis was conducted with package: “TwoSampleMR”, “VariantAnnotation”, and “ieugwasr”. Five methods were carried out in MRanalysis: “MR Egger, [29]” “Weighted median, [30]” “Weighted mode. [30]” “Inverse variance weighted (IVW), [31]” and “Simple mode. [32]” Herein, we mainly focus on the results of IVW, which provided the most precise and robust estimates when three key assumptions are met. The heterogeneity and the pleiotropy test were performed with Cochran’s Q statistic. and MR-Egger intercept test, respectively [33]. Moreover, for detecting and correcting horizontal pleiotropy, the MR pleiotropy residual sum and outlier method (MR-PRESSO) was executed with the “MR-PRESSO” package [34]. For exploring whether the analysis was driven or biased by a single SNP, a leave-one-out analysis was also executed.

### Availability of data and materials

The data using in the study were gathered from GWAS summary data (https://gwas.mrcieu.ac.uk/; LC: Ebi-a-GCST90018875; SCLC: Finn-b-C3_SCLC; NSCLC: Finn-b-C3_LUNG_NONSMALL; 731 ICTs: Ebi-a-GCST0001391 to Ebi-a-GCST0002121).

## Supplementary Material

Supplementary Table 1

Supplementary Table 2

Supplementary Table 3
